# Development of a Choline Database to Estimate Australian Population Intakes

**DOI:** 10.3390/nu11040913

**Published:** 2019-04-23

**Authors:** Yasmine Probst, Vivienne Guan, Elizabeth Neale

**Affiliations:** 1 Smart Foods Centre, School of Medicine, University of Wollongong, Wollongong, NSW 2522, Australia; xg885@uowmail.edu.au (V.G.); elizan@uowmail.edu.au (E.N.); 2 Illawarra Health and Medical Research Institute, Wollongong, NSW 2522, Australia

**Keywords:** food composition, national survey, food intake, choline, informatics

## Abstract

The AUSNUT 2011–13 food composition database was expanded to include Australian choline values. The development began with a systematic literature review of published studies. Analytical data from the food studies were extracted and aligned with their equivalent AUSNUT food identification code. Global food composition databases containing choline values were matched to the remaining AUSNUT food codes, following the FAO INFOODS food matching guidelines, including adjustments for moisture and protein composition. Composite foods, and not further-specified foods, were developed using the Food Standards Australia New Zealand (FSANZ) recipe files. The completed choline database was then employed to analyse the Australian National Nutrition and Physical Activity Survey 2011–12, with population and sampling weightings applied. Survey respondents were classified into categories based on their level of choline intake and compared with the Australian Adequate Intake levels. Food sources of intake were also explored. Multiple linear regression models were developed for food group contributors to choline intake. Mean choline intakes varied from 151.50 mg for pregnant 14–18 years old, to 310.54 mg for 19–64 year old males. Less than 10% of the population by age and gender were achieving the Adequate Intake for choline. Eggs and their contributing food groups were the top ranked food sources of choline for the population.

## 1. Introduction

Choline is an essential nutrient for humans, though it can also be synthesised endogenously by the human liver [1,2]. Choline is thought to belong to the B-group complex of vitamins. Discovered in 1962, choline was isolated by the boiling of pig bile following a renewed interest in its deficiency status [2]. These early studies concluded that humans did not need to eat choline from food sources. Later research uncovered important links between choline transport via the placenta [2]. These study findings also uncovered an increased need for choline in certain population groups, e.g., males and post-menopausal females, resulting in the development of dietary guidance for this nutrient [3]. It is speculated that oestrogen plays a role in the biosynthesis of choline [4]. During pregnancy, however, females with low levels of vitamin B12 or folic acid may be at risk of inadequate choline, due to its role in methyl group metabolism [5]. Choline donates methyl groups to homocysteine to form methionine in methyl group metabolism. DNA methylation increases for cell differentiation and organogenesis during embryogenesis and early postnatal life [5]. The literature suggests that maternal choline supplementation is positively related to offspring neurocognitive outcomes [6,7].

Given DNA methylation is an epigenetic modification which regulates the patterns of gene expression, DNA methylation patterns are critical in mammalian development and later physiological function in adulthood [8,9]. Additionally, choline is a precursor to compounds that maintain human health, including cell membrane constituents (phospholipid and sphingomyelin) and neurotransmitters (acetylcholine) [5]. Choline also plays a role in lipid and cholesterol transport [5]. Studies suggests that low intakes of choline have been linked to cardiovascular disease [10], neurological disorders such as Alzheimer’s disease [11] and fatty liver disease [12]. 

To determine levels of consumption within a population, suitable food composition data is required. Food composition databases are used in practice across a variety of areas, including food policy formulation, food labelling and dietary assessment [13]. Food composition databases allow for food information reported by population groups during a dietary assessment to be translated into nutrient information. Databases also create a framework for food grouping systems that can be used for dietary pattern analyses in relation to the nutrients [14]. Choline and its esters are widely found in foods. Although choline can be formed in the liver, the majority of people need to consume choline from dietary sources to meet their requirements [3]. While many studies have used data from existing food composition databases, it is imperative that regionally-specific food composition data is used, due to the variation in food harvesting, processing and preparation conditions in different countries [15]. Following the development of dietary guidance values for choline during the early 2000s, the United States Department of Agriculture sought to create choline values for its standard reference food composition database. This development also created retention factors for losses of choline due to cooking, with variations of between 70 and 100% retention. Where no data were available, choline retention factors were imputed from other B-group vitamins due to their similarities [16]. Such developments have provided an impetus for other countries to create data for their food supply, though until recently, no data was available for Australian foods. The aim of this study was to develop an Australian choline database, and to apply this database to Australian population data to identify choline consumption patterns by age and gender.

## 2. Materials and Methods 

### 2.1. Development of an Australian Choline Database

In order to explore choline consumption from respondents to the 2011–13 Australian Health Survey [17], foods listed in the AUSNUT 2011–13 food composition database [18] needed to have individual choline values. In the absence of resources to analyse foods chemically under laboratory conditions, this study sourced published studies and global food composition databases containing choline values. These values were compared with nutrient data for Australian foods, using standardised approaches reported previously for other food components. Data were then converted as required to create a ‘choline database’ of values aligned to each AUSNUT 2011–13 food type. To develop this database, a process adapted from an existing database was followed [19,20]. 

#### 2.1.1. Systematic Literature Review 

A systematic literature review was conducted. The review is reported according to the Preferred Reporting Items for Systematic Review and Meta-Analyses (PRISMA) guidelines and was required to source existing published data for Australian food types. The review answered the questions ‘Which global food composition databases report values for choline in foods?’ and ‘Do the foods reported relate to those in the Australian food supply?’ As the purpose of the review was to source choline values only, the review was not registered with the International Prospective Register of Systematic Reviews (PROSPERO).

Search strategy: A systematic search was performed using the following scientific databases: Scopus, Web of Science, PubMed and Science Direct. The search was undertaken 3-4 October 2018, including studies from January 2008 through to September 2018. This period was applied, due to the progress in choline data during this time. Direct chemical analytical studies using foods to create food composition data or to assess dietary intake were included in the review.

Due to a close physiological interaction within the body the search terms ‘choline’ OR ‘betaine’ were used as these compounds are metabolically similar, and often reported together.

Eligibility criteria: Analytical food studies were included if the (1) choline and betaine content of foods were reported (2) food items were listed in the AUSNUT 2011–13 food composition database (3) dietary intake estimation studies used food composition data based on published food composition databases other than the USDA choline and betaine databases, published and/or unpublished literature and/or analytical experiments. 

Studies were excluded if they (1) used a metabolomic approach or biomarkers, to examine choline and betaine content (2) aimed to optimise the nutrients composition of foods; aimed to develop or validate new analytical methods (3) used USDA choline and betaine databases to estimate dietary intake (4) were published in languages other than English (5) analysed foods not matched to the food items found in the AUSTNUT 2011-13 food composition database. 

Data extraction: The titles and abstracts of articles were screened to identify the most relevant studies. The full-text articles were reviewed by assessing eligibility related to the inclusion and exclusion criteria. Data related to the country of origin, food types, method of analysis and amount and type of choline were extracted from the included studies. Reference lists of all included studies were hand searched.

#### 2.1.2. Assigning Food Composition Data

Sourcing global food composition databases: Existing food composition databases were extracted from the literature search outlined in 2.1.1. Where data for choline was still required for Australian foods, these were sourced from existing choline databases based upon matches by food type, origin, botanical name, species and/or cooking method. To aid this process, foods were aligned to the food groups of the USDA choline database [21] as this was deemed to be the most comprehensive existing database. 

Matching food items: Following the identification of existing databases, the FAO INFOODS guidelines for food matching were applied [22]. The matching guidelines provide criteria and a confidence code for each food, based on how well it matched with another food item. The confidence codes begin with an A for a high quality exact food match, through to a D for a weak food match. The matching file was originally developed for a selection of foods for the purposes of dietary assessment analysis, though it was adopted for this study, given the common decision-making elements needing to be employed. The process of matching was undertaken for food items which were matched to one single food item or to a recipe. 

Creating composite foods: For composite foods or those created using a recipe, the Food Standards Australia New Zealand (FSANZ) recipe file was used [23]. A total weight change factor of food/ingredients in the recipe was applied during the calculation, which accounted for moisture losses or gains from cooking. Equivalence weights from the recipe file were used to create a representative composite food calculated from the single foods (ingredients) in the database.

Quality assurance checking: In the final database, all foods were rank-ordered, and range checks were applied to the choline values to determine any incorrect or outlier data points. All calculations undertaken with the database development were also checked in duplicate to reduce human error. Prior to it application to the population intake data, a process of source data verification was also undertaken using a 10% random sample of the completed database. Any errors were corrected accordingly.

### 2.2. Australian Choline Intakes Using the Australian Health Survey

Data from the developed choline database were aligned with the intake summaries of the National Nutrition and Physical Activity Survey (NNPAS) 2011–12, from the wider Australian Health Survey 2011–13 [17]. Data management was undertaken using Microsoft Excel. Day 1 and day 2 intake data was normalised for the purpose of descriptive analyses. This was conducted using the Multiple Source Method (2017, Department of Epidemiology of the German Institute of Human Nutrition Potsdam-Rehbrücke, https://msm.dife.de/), by a shrinkage technique applied to the residual of a regression model, and was used to represent habitual intakes of the population. 

All statistical analyses were undertaken using STATA IC (release 15, 2017. Stata Statistical Software: College Station, TX, USA: StataCorp LLC). Both sampling and population weightings were applied to the dataset [24]. Intakes were presented by age group and gender as mean and standard errors. These values were compared with the Australian Adequate Intake (AI) values for choline by age group and gender [1]. Age groups were considered as follows: Children 2–3, 4–8, 9–13, 14–18 years, adults 19–64, 65–85 years, and females of childbearing age, which was considered to be 16–44 years, based on the nutrient reference value categories, and alignment with analyses conducted by the Australian Bureau of Statistics.

Day 1 intake data for the population were then analysed to determine the top 20 food sources for choline intake in the Australian population. These data were based on intakes for the total population, as well as the proportion of total choline intake for persons consuming choline. Values were presented based on the AUSNUT 2011–13 three digit level food groupings, showing the average choline intakes for the proportion of the population consuming each food group. Key food sources for the age and gender groups and for women of child-bearing age were determined. 

Regression models were developed to determine the predictive food sources of choline intakes in the Australian population. Quartiles of choline intake were reported. Multiple linear regression models were employed for intakes of the food groups in grams. Contribution to total energy intake, gender, age group, physical activity and level of education were considered as independent variables to examine the contribution of the food groups to the inter-individual variation in total choline intakes. The infant formula and infant foods food groups were excluded from the analysis due to the age groups used in the NNPAS. The regression outcomes represent the change in total choline intakes per 100 g increments of the intake of the corresponding food group. A value of *p* < 0.05 was set to indicate statistical significance.

## 3. Results

### 3.1. Australian Choline Database

From the systematic literature review, studies using choline data were identified for Australia, USA, Alberta, New Zealand, Japan and Canada. The majority of studies used the USDA database for analysis of intake data, including population level consumption surveys. For the development of the choline database, the following stages were employed:

1. Literature sources were obtained from three published studies [25,26,27].
2. USDA database for the choline content (4530 foods).

As the data from the USDA database does not align with the Australian food supply, a number of steps needed to be undertaken to ensure that matches of foods were of the highest quality. For this process:

- Substitutions were made based on similar energy contributions and conceptual descriptions of food appearance (2576 substitutions).
- Recipe calculations were used (105 new recipes were created).
- Analytical data was used for 26 data points. This included several varieties of pulses (chickpeas, black beans and lima beans), shellfish and seafood (shrimp).
- In total, 2707 foods matches came from a USDA source

3. In total, 5597 foods were aligned with a choline value (97.51% of the AUSNUT 2011-13 database). The database can be sourced by contacting the corresponding author of this study. Where a zero value was provided, no values were identified. While traditionally zero values are reserved for foods without any trace amounts of a nutrient i.e., theoretical zero, for the purpose of the subsequent analyses in this study, zero was used. Many of these foods may also contain trace or values below the limits of quantification.
4. Betaine and choline were commonly reported together. Where this occurred, betaine data was also added to the database (3910 foods in total).

### 3.2. Australian Population Intakes of Choline

Total population analyses of the NNPAS were completed for *n* = 12,153 respondents, which equaled a population-weighted sample of 21,526,456 persons. Of this data, 5702 were male and 6451 female. In total, 2048 respondents were 18 years of age and below, and 9115 were over the age of 19 years. Of the sample used in this study, a population equivalent of 4,073,867 females of childbearing years were identified. 

The intakes of the survey respondents ranged from 151 mg for younger (14–18 years) pregnant females, through to 311 mg for adult males between 19 and 64 years of age. The mean intake for the population as a whole was 265.18 ± 1.3 mg. Intakes by gender, including respondents who were equal to or above the AI values, are shown in Table 1. The results show that more than two thirds of 2–3 year old children were achieving the AI, though this declined to only one in five by 4–8 years of age, and declined further during adolescence. For the adults, less than 4% of the respondents were reaching the AI. The AI values increase to 440 mg during pregnancy, and 550 mg during lactation, with less than 1% of females achieving this level. 

#### 3.2.1. Food Sources of Choline for the Australian Population

**Figure 1:**
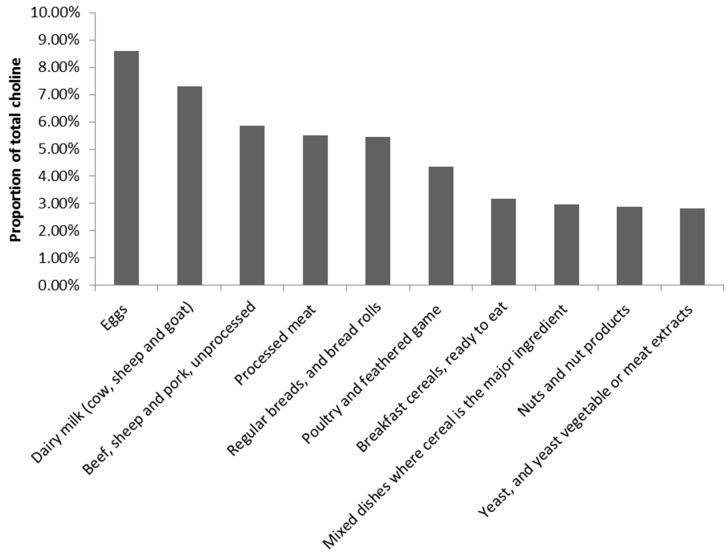
Food group contributors to Australian choline intakes ^1^. ^1^ Food groups equate to 48.83% of total choline intake. The remaining 51.17% of contributions were from food groups including, but not limited to, cheese, dishes where egg is the major component, mixed dishes, where poultry or feathered game is the major component, potatoes, cakes, muffins, scones, cake-type desserts, missed dishes, where beef, sheep or mammalian game is the major component, chocolate and chocolate-based confectionery, pastries, fin fish, fishes where vegetable is the major component. The top 20 food groups equate to 68.45% of the total choline intake.

For the population overall, the highest contributing food group to choline intake was the eggs food group, followed by dairy milk (Figure 1). This pattern shifted slightly when analysed by gender and age groups. Dishes where eggs are the major ingredient remained the leading contributor to choline intakes for all child respondents in the survey, while the eggs food group varied between the second and third-ranked contributor (see Supplementary Material). 

Greater shifts in food group contributions became evident for the adult analyses. The same five food groups held ranks one to five for both males and females. Mammalian game meat moved to the top-ranked contributor to choline intake, while the second- and third-ranked food groups were egg products and dishes, followed by eggs. This was followed by mixed dishes with beef, sheep, pork or mammalian game, and in fifth position beef, sheep or pork unprocessed meat. (see Supplementary Material). While these differences affected the rank order of the food groups, it can be seen that the average choline contribution for females did not vary substantially between the top two ranked food groups, while the difference was larger for adult males (~80 mg). 

When the data was further filtered for females of childbearing age, the top-ranked food group was replaced with other sea and freshwater food for pregnant females, followed by egg products and dishes. Egg products and dishes and eggs formed the top two food groups for choline for lactating females.

#### 3.2.2. Food Groups Associated with High Choline Intake

Using each of the major food groups, regression models were created to determine key predictors for choline intakes for the Australian population. The strongest predictor of choline intake was the meat, poultry and game products and dishes food group, explaining 22.62% of the variance in choline intakes (Table 2). This was closely followed by egg products and dishes (14.49%) (Table 3), cereal-based products and dishes (11.16%) (Table 4), alcoholic beverages (10.80%), milk products and dishes (9.30%), vegetable products and dishes (8.34%) and cereal products and dishes (7.48%) (all, *p* < 0.05). The meat products and dishes regression model was significantly influenced by gender and level of education, while the egg products and dishes and the alcoholic beverages models appeared to be influenced by the energy intake of the respondents.

## 4. Discussion

The present findings, the first Australian representative intake estimates of choline at a national level, suggest that the estimated choline intakes in Australia (265.18 ± 1.3 mg; 151–311 mg) are below the values reported in the current literature though there is considerable variation in the methods applied between countries. For Australians aged 19–64 years, the mean choline intakes were 310.54 mg/day and 247.65 mg/day for males and females, respectively. The estimated choline intake based on the National Health and Nutrition Examination Survey 2013–14 in the USA reported mean choline intakes between 359 mg/day to 426 mg/day for males aged 20–69 years, and 275 mg/day to 296 mg/day for females [28]. The European adult male intake estimates ranged from 357 mg/day to 468 mg/day (18–65 years), and 291 mg/day to 374 mg/day for females [29]. The Australian intakes of choline for childbearing females and women during pregnancy and lactation were also less than the intakes revealed from the published studies. For example, the estimated choline intakes of females aged 18–40 years was 316 mg/day in New Zealand [30]. During pregnancy in Canada it was 347 mg/day [31], and Latvian values ranged from 336mg/d to 356 mg/day [29]. However, the comparability of these choline intakes should be interpreted with caution. Each of these values are higher than the reported Australian values, though this may be due to the use of borrowed food composition data. Even though the USDA choline food composition database was applied in these studies [28,29,30], different releases of the databases may contribute to the discrepancy between intake estimates. As the USDA choline database is the most comprehensive database for choline in foods globally, many countries will apply this data to the reported food intakes of their own country. Best practice recommendations for food intake assessment are for the use of regionally-appropriate food composition data when analysing food intakes. This was applied within the current study though a large proportion of the data was still borrowed, due to the limited Australian studies. This reliance on borrowed data stresses the need for more studies focused on the analysis of foods for their choline composition. These compositional analyses should align with the AOAC analytical methods, and be suited to the food matrix that is being analysed. By growing the number of Australian studies with choline content analyses, researchers can become less reliant on borrowed data, and lessen the need to create assumptions when matching a reported food type to a food composition value. For example, this was evident in this study for the mammalian game meats food group, which includes kangaroo meat. No published studies were identified, and borrowed data was required for the database development. Kangaroo meat is unique to Australia and, therefore, the closest equivalent meat type needed to be matched to the kangaroo values in the Australian AUSNUT 2011-13 food composition database, as suggested by the FAO INFOODS food matching guidelines [22]. Subsequently, this may also impact the food group level outcomes as outlined below.

Another difference between the studies reporting population level choline intakes may be the dietary assessment method used in the survey, and the method of analysis. Studies have shown differences in data between the use of day 1, and day 2, as well as an average of days 1 and 2, and also the use of a regression model to represent usual intakes. This study applied usual intake analyses to the NNPAS data, which considered the reporting of both days 1 and 2. There are fluctuations around individual intakes reflecting true eating habits in free-living conditions. Thus, estimation of usual dietary intake will create a value that is more representative of habitual intakes, in comparison to using only one day of data. 

Australian intakes for both genders and across the majority of age groups were not aligned with the AI levels. Similar trends of suboptimal choline intake were also found in the US population for different age groups, suggesting that only 11% of the US population aged ≥2 years met the AI for choline [32]. 

Although the literature suggested that dietary choline intakes were largely contributed by the variation of energy intake [33], the developed AI levels were largely from experimental studies, and were based on 7 mg/kg body weight. These levels were based on a 76 kg male and a 61 kg female [1] to determine the targets. Due to the high rates of obesity within the Australian population, it is anticipated that a 7 mg/kg body weight intake level would not be achieved. The pregnancy AI values for choline add an additional 11 mg/day, based on the assumption that no additional choline is produced by the foetus or the placenta. The demands of choline during pregnancy and lactation increase [5]. Choline, as a supply of methyl groups plays a critical role in stem cell proliferation and apoptosis, thereby influencing the structure and function of the brain and spinal cord in relation to a risk of neural tube defects and the lifelong memory function of the foetus and infant [5]. The added requirements for choline during pregnancy are of a greater concern, given the extremely low levels of choline for the pregnant and lactating females in this study. Despite this, two in every three reported intakes for infants met the AI levels. This may be related to parental influence in eating patterns during this life stage, and the relatively small amounts of food required. The reporting of infant intakes by parents may also be influenced by a desirability bias, whereby the parent may unconsciously increase the amount of particular foods reported. 

The major food source for choline in this study was found to be egg products and dishes for children and lactating females. This shifted for the adult intakes, though egg-related food groups continued to be within the top five ranked food groups. Foods derived from animal sources, such as eggs, meat, fish and milk, generally contain more choline than plant-based foods, such as grain, vegetable and fruit in per unit weight [25,26,27,34]. In the USA, the major food sources of choline intake were meat, poultry and fish, grain-based mixed meals, dairy and eggs [28]; where meat, milk, grain, eggs and their derived products, composite dishes and fish were the main contributors in European countries [29]. The foods contributing to choline intake in New Zealand were eggs, red meat, milk and bread [30]. Outcomes of this study were most comparable with those of New Zealand. Interestingly, due to the detailed food group categorisation in the present analyses, the top-ranked food group position for adults 19–64 years of age was found to be from the mammalian game meat food group, contributed by kangaroo meat, which is unique to Australian intakes. As outlined above, kangaroo meat did not have analysed choline values in the food composition database, requiring food data to be matched and borrowed from the USDA. Further, only a small proportion of the population reported consuming kangaroo meat, but the portions consumed were larger (~210g) by comparison to the portions consumed from the eggs food group (~90g) for example. Similarly, for pregnant females the other sea and fresh water foods food group contributions had a substantial impact on choline intakes. Again, this was reported by a small proportion of the population, likely due to pregnancy intake guidelines for this population group [35]. 

While some similarities were seen between the Australian food group contributions and other countries, the discrepancy between the Southern and Northern hemisphere findings may be due to the level of detail of food items that were reported. Instead of grouping food items based on the dominant nutrients or ingredients, for example cereal and meat, the food items in the present analyses were categorised on the basis of similar animal/plant species or family, or sharing similar cooking methods and derived from a nested hierarchical food group classification system [36]. The present study provides more detail on the actual foods consumed by the respondents in the NNPAS, which may have a more practical focus for dietary recommendations. This level of detail may also be indicative of the differences between the published intake studies for choline to date. Recipe calculations were also performed in the present study. Eggs are often used as an ingredient item in a number of dishes which may be categorised within food groups other than the egg products, and dishes food group. Creating a method to quantify the use of eggs within other food items of the food composition tables will allow for other smaller egg contributions to be included. Such an analysis at a recipe and food level may in future provide a more accurate estimate of total population intakes of eggs, which may also be apparent for milk when used as an ingredient. For the purpose of this study, however, the choline content would have been accounted for within each of the food groups used in the analyses.

There are a number of limitations to this study. Development of the choline database required substantial use of professional judgement. While quality assurance measures were implemented, some matches may require local knowledge of the food supply to ensure the matches are correct. Further, while population intakes were based on nationally-representative intake data, it was based on self-reported data only, which may be susceptible to bias. Adjustments of intake were also not made for the analyses reported in this study. 

Food group predictors of Australian population choline intakes have not been reported in the published literature to date. This study found that meat, poultry and game products and dishes were the main predictive food group for choline intake, followed by egg products and dishes and cereal-based products and dishes. While these food groups were the primary predictors for choline intake, they predicted a combined 52.27% variation in choline intakes. The remaining predictors were weaker, and spanned across a wide range of food groups. These food groups were significantly influenced by covariates of the model, namely the gender, level of education and the energy intake of the respondents. These factors have also been associated with diet quality, whereby females compared with males [37] and those with higher levels of education [38] are reported to consume a higher quality diet.

In conclusion this study has provided preliminary data for a choline database to be used with Australian foods. It has aligned existing published values with entries of the Australian food composition survey database and applied these to recent consumption data to estimate population intakes. This has provided insight into food-based sources of choline for Australia. The data for the choline content of Australian food items should be updated over time to provide more accurate estimates of intake, particularly as newer consumption survey data becomes available.

## Figures and Tables

**Table 1:** Choline intakes for the Australian population (*n* = 12153) by gender.

Choline, mg	*n*	Population Equivalent. ^1^	Mean	SE	95% CI	Q1	Q2	Q3	Q4	AI ^2^, Mg/Day	Intake ≥ AI ^2^	
											% (*n* =)	Population Equivalent. ^1^
**Total intake**	12,153	21,526,456	265.18	1.30	262.58–267.77	0.00–198.09	198.09–249.90	249.90–315.64	315.64–578.57	NA	-	-
**Children**												
2–3 years	464	561,399.4	185.36	3.43	178.50–192.22	0.00–148.97	148.97–178.91	178.91–212.40	212.40–380.50	200	67.03% (311)	382,817.22
4–8 years	789	1,372,102	203.37	2.92	197.530–209.21	0.00– 154.78	154.78–189.68	189.68–232.29	232.29–411.17	250	20.03% (158)	261,468.33
**Male**												
9-13 years	392	770,917.54	244.17	5.17	233.82–254.52	0.00–195.99	195.99–240.21	240.21–284.74	284.74–464.58	375	7.14% (28)	54,114.994
14–18 years	403	660,624.57	275.37	6.24	262.89–287.85	0.00–215.43	215.43–251.69	251.69–327.02	327.02–569.06	550	1.99% (8)	11,233.998
19–64 years	3372	6,899,404	310.54	2.49	305.56–315.51	0.00–232.12	232.12–293.67	293.67–367.42	367.42–676.34	550	3.74% (126)	246,086.08
65–85 years	910	1,374,767	281.29	3.28	274.73–287.86	0.00–222.38	222.38–271.42	271.42–329.75	329.75–541.46	550	1.21% (11)	12,317.192
**Female**												
9–13 years	395	755,088.81	228.06	4.50	219.05–237.06	0.00–175.76	175.76–222.25	222.25–267.81	267.81–443.44	375	4.30% (17)	27,728.827
14–18 years	367	645,741.25	229.02	6.28	216.46–241.59	0.00–173.31	173.30–214.51	214.51–274.77	274.77–493.26	400	1.91% (7)	10,866.495
Pregnant (14–18 years)	2	4,220.548	151.50	26.94	97.60–205.40	-	-	-	-	415	0	0
19–64 years	3640	6,521,097	247.65	1.92	243.82–251.49	0.00–191.10	191.10–236.41	236.41–291.21	291.21–510.75	425	3.32% (121)	237,729.29
Pregnant (19–50 years)	116	213,030.18	252.91	10.32	232.25–273.57	0.00–193.15	193.15–250.72	250.72–304.46	304.46–428.67	440	0.86% (1)	1,658.7132
Lactating (19–50 years)	110	202,643.04	253.86	8.01	237.84–269.88	0.00–195.44	195.44–256.93	256.93–295.00	295.00–478.55	550	0.91% (1)	166.809503
65–85 years	1193	1,545,421	249.00	2.59	243.80–254.18	0.00–198.49	198.49–239.17	239.17–287.35	287.35–479.60	425	3.02% (36)	46,117.23
**Childbearing age (16–44 years) ^3^**	2210	4,073,867	243.63	2.09	246.01–254.71	0.00–187.43	187.43–233.12	233.12–290.76	290.76–511.08	425, 19–44 years ^4^	4.75% (102, 19–44 years ^5^)	195,107.54 19–44 years ^6^

^1^ Population equivalence determined using weighting factors, ^2^ Adequate intake, ^3^ Excluding pregnant and breastfeeding women, ^4^ 400 for 16–18 years, ^5^ 105 for 16–18 years, ^6^ 3,050.9551 for 16–18 years.

**Table 2:** Linear regression outcomes of the meat, poultry and game products and dishes food group for choline intake in the 2011–12 NNPAS ^1^ (*n* = 21,526,456).

	Coefficient	Jackknife Standard Error	T	*P* > |t|
Choline	48.245	1.388	34.76	0.000
Energy	−0.001	0.001	−0.86	0.378
Physical activity ^2^	0.542	0.715	0.76	0.451
Gender	−9.131	2.174	−4.20	0.000
Age	−0.098	0.071	−1.38	0.172
Education level ^3^	2.174	0.563	3.86	0.000

^1^ National Nutrition and Physical Activity Survey; ^2^ Measured in ‘levels’ where Level 1 indicates ‘high’ level of physical activity and Level 5 indicates ‘sedentary’ activity.; ^3^ Measured in ‘levels’ where Level 1 indicates highest level of non-school education, and Level 5 indicates lowest level.

**Table 3:** Linear regression outcomes of the egg products and dishes food group for choline intake in the 2011–12 NNPAS ^1^ (*n*= 21,526,456).

	Coefficient	Jackknife Standard Error	T	*P* > |t|
Choline	30.558	1.374	22.25	0.000
Energy	−0.005	0.001	−7.73	0.000
Physical activity ^2^	−1.128	0.640	−1.76	0.083
Gender	2.114	1.712	1.23	0.222
Age	0.023	0.534	0.42	0.673
Education level ^3^	0.321	0.447	0.72	0.475

^1^ National Nutrition and Physical Activity Survey; ^2^ Measured in ‘levels’ where Level 1 indicates ‘high’ level of physical activity and Level 5 indicates ‘sedentary’ activity.; ^3^ Measured in ‘levels’ where Level 1 indicates highest level of non-school education, and Level 5 indicates lowest level.

**Table 4:** Linear regression outcomes of the cereal-based products and dishes food group for choline intake in the 2011–12 NNPAS ^1^ (*n* = 21,526,456).

	Coefficient	Jackknife Standard Error	T	*P* > |t|
Choline	2.882	0.917	3.14	0.003
Energy	0.008	0.001	12.53	0.000
Physical activity ^2^	0.991	0.534	1.86	0.069
Gender	–0.402	1.403	–0.29	0.776
Age	0.693	0.048	–8.77	0.000
Education level ^3^	-6.667	0.335	2.07	0.043

^1^ National Nutrition and Physical Activity Survey; ^2^ Measured in ‘levels’ where Level 1 indicates ‘high’ level of physical activity and Level 5 indicates ‘sedentary’ activity.; ^3^ Measured in ‘levels’ where Level 1 indicates highest level of non-school education, and Level 5 indicates lowest level.

## Supplementary Materials

The following are available online at https://www.mdpi.com/2072-6643/11/4/913/s1, Figure S1: PRISMA flow diagram of literature search, Figure S2: PRISMA Checklist, Table S1: Proportion of young children intakes of choline by food group, Table S2: Average intakes for male consumers of choline by food group, Table S3: Average intakes for female consumers of choline by food group, Table S4: Average intakes for pregnant and lactating female consumers of choline by food group.
