# Supplementary material for: Development of a Choline Database to Estimate Australian Population Intakes
Source: Nutrients. 2019 Apr 23;11(4):913. doi: 10.3390/nu11040913 (PMC6521034; doi:10.3390/nu11040913)
Supplement: Supplementary file 1 [file nutrients-11-00913-s001.zip › nutrients-477121-supplementary-figure S1,Tables.docx]

**Supplementary Material**


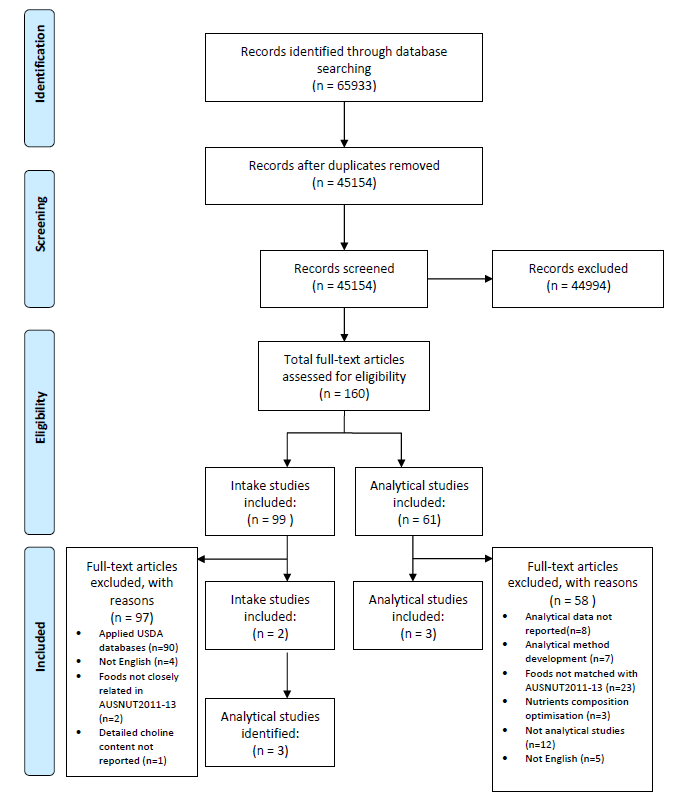


**F**igure S1: PRISMA flow diagram of study selection

**Table S1.** Proportion of young children intakes of choline by food group

|  | 2-3 years | |  | 4-8 years | |  |
| --- | --- | --- | --- | --- | --- | --- |
| **Rank** | **ID** | **Food group** | **Mean choline (mg)** | **ID** | **Food group** | **Mean choline (mg)** |
| 1 | 172 | Dishes where egg is the major ingredient | 137.38 | 172 | Dishes where egg is the major ingredient | 189.24 |
| 2 | 171 | Eggs | 102.77 | 171 | Eggs | 110.32 |
| 3 | 187 | Mixed dishes where beef, sheep, pork or mammalian game is the major component | 74.08 | 181 | Beef, sheep and pork, unprocessed | 89.16 |
| 4 | 181 | Beef, sheep and pork, unprocessed | 73.47 | 151 | Fin fish (excluding commercially sterile) | 83.12 |
| 5 | 183 | Poultry and feathered game | 66.77 | 187 | Mixed dishes where beef, sheep, pork or mammalian game is the major component | 81.56 |
| 6 | 151 | Fin fish (excluding commercially sterile) | 55.18 | 182 | Mammalian game meats | 78.12 |
| 7 | 191 | Dairy milk (cow, sheep and goat) | 54.78 | 183 | Poultry and feathered game | 73.75 |
| 8 | 201 | Dairy milk substitutes, unflavoured | 53.35 | 323 | Infant foods | 66.07 |
| 9 | 205 | Soy-based yoghurts | 50.34 | 205 | Soy-based yoghurts | 66.06 |
| 10 | 185 | Sausages, frankfurts (what frankfurters are called in Australia) and saveloys | 49.82 | 152 | Crustacea and molluscs (excluding commercially sterile) | 62.74 |
| 11 | 189 | Mixed dishes where poultry or feathered game is the major component | 49.25 | 185 | Sausages, frankfurts (what frankfurters are called in Australia) and saveloys | 61.18 |
| 12 | 155 | Fish and seafood products (homemade and takeaway) | 47.13 | 202 | Dairy milk substitutes, flavoured | 60.46 |
| 13 | 156 | Mixed dishes with fish or seafood as the major component | 45.61 | 156 | Mixed dishes with fish or seafood as the major component | 57.60 |
| 14 | 251 | Mature legumes and pulses | 45.36 | 189 | Mixed dishes where poultry or feathered game is the major component | 56.41 |
| 15 | 198 | Flavoured milks and milkshakes | 39.48 | 135 | Mixed dishes where cereal is the major ingredient | 52.27 |
| 16 | 135 | Mixed dishes where cereal is the major ingredient | 38.88 | 301 | Formula dietary foods | 50.58 |
| 17 | 252 | Mature legume and pulse products and dishes | 34.30 | 184 | Organ meats and offal, products and dishes | 50.33 |
| 18 | 133 | Cakes, muffins, scones, cake-type desserts | 33.70 | 198 | Flavoured milks and milkshakes | 47.97 |
| 19 | 211 | Soup, homemade from basic ingredients | 32.15 | 211 | Soup, homemade from basic ingredients | 45.44 |
| 20 | 321 | Infant formulae and human breast milk | 31.15 | 134 | Pastries | 43.39 |

**Table S2.** Average intakes for male consumers of choline by food group

|  | 9-13 years | |  | 14-18 years | |  | 19-64 years | |  |
| --- | --- | --- | --- | --- | --- | --- | --- | --- | --- |
| **Rank** | **ID** | **Food group** | **Mean choline (mg)** | **ID** | **Food group** | **Mean choline (mg)** | **ID** | **Food group** | **Mean choline (mg)** |
| 1 | 172 | Dishes where egg is the major ingredient | 182.96 | 172 | Dishes where egg is the major ingredient | 156.25 | 182 | Mammalian game meats | 250.83 |
| 2 | 171 | Eggs | 155.86 | 171 | Eggs | 120.49 | 172 | Dishes where egg is the major ingredient | 172.72 |
| 3 | 184 | Organ meats and offal, products and dishes | 128.10 | 189 | Mixed dishes where poultry or feathered game is the major component | 107.76 | 171 | Eggs | 171.94 |
| 4 | 181 | Beef, sheep and pork, unprocessed | 120.49 | 152 | Crustacea and molluscs (excluding commercially sterile) | 107.72 | 187 | Mixed dishes where beef, sheep, pork or mammalian game is the major component | 164.36 |
| 5 | 201 | Dairy milk substitutes, unflavoured | 118.21 | 215 | Soup, commercially sterile, prepared from condensed or sold ready to heat | 104.14 | 181 | Beef, sheep and pork, unprocessed | 148.40 |
| 6 | 187 | Mixed dishes where beef, sheep, pork or mammalian game is the major component | 108.70 | 181 | Beef, sheep and pork, unprocessed | 99.82 | 189 | Mixed dishes where poultry or feathered game is the major component | 138.69 |
| 7 | 189 | Mixed dishes where poultry or feathered game is the major component | 107.06 | 183 | Poultry and feathered game | 96.86 | 184 | Organ meats and offal, products and dishes | 137.73 |
| 8 | 183 | Poultry and feathered game | 104.83 | 187 | Mixed dishes where beef, sheep, pork or mammalian game is the major component | 88.55 | 156 | Mixed dishes with fish or seafood as the major component | 135.01 |
| 9 | 151 | Fin fish (excluding commercially sterile) | 100.77 | 252 | Mature legume and pulse products and dishes | 84.04 | 183 | Poultry and feathered game | 134.45 |
| 10 | 155 | Fish and seafood products (homemade and takeaway) | 79.33 | 156 | Mixed dishes with fish or seafood as the major component | 79.56 | 155 | Fish and seafood products (homemade and takeaway) | 127.04 |
| 11 | 135 | Mixed dishes where cereal is the major ingredient | 74.64 | 135 | Mixed dishes where cereal is the major ingredient | 74.17 | 151 | Fin fish (excluding commercially sterile) | 124.27 |
| 12 | 185 | Sausages, frankfurts (what frankfurters are called in Australia) and saveloys | 74.58 | 196 | Custards | 67.34 | 153 | Other sea and freshwater foods | 119.23 |
| 13 | 152 | Crustacea and molluscs (excluding commercially sterile) | 72.80 | 301 | Formula dietary foods | 67.05 | 291 | Beers | 105.78 |
| 14 | 252 | Mature legume and pulse products and dishes | 63.68 | 198 | Flavoured milks and milkshakes | 64.41 | 185 | Sausages, frankfurts (what frankfurters are called in Australia) and saveloys | 104.64 |
| 15 | 134 | Pastries | 61.47 | 155 | Fish and seafood products (homemade and takeaway) | 56.48 | 205 | Soy-based yoghurts | 100.22 |
| 16 | 156 | Mixed dishes with fish or seafood as the major component | 61.11 | 126 | Breakfast cereals, hot porridge style | 56.40 | 152 | Crustacea and molluscs (excluding commercially sterile) | 93.02 |
| 17 | 198 | Flavoured milks and milkshakes | 57.20 | 251 | Mature legumes and pulses | 54.02 | 135 | Mixed dishes where cereal is the major ingredient | 88.98 |
| 18 | 191 | Dairy milk (cow, sheep and goat) | 53.55 | 134 | Pastries | 52.45 | 198 | Flavoured milks and milkshakes | 86.03 |
| 19 | 126 | Breakfast cereals, hot porridge style | 53.43 | 151 | Fin fish (excluding commercially sterile) | 51.77 | 188 | Mixed dishes where sausage, bacon, ham or other processed meat is the major component | 84.51 |
| 20 | 211 | Soup, homemade from basic ingredients | 51.51 | 211 | Soup, homemade from basic ingredients | 48.00 | 216 | Soup, purchased ready to eat | 84.30 |

**Table S3.** Average intakes for female consumers of choline by food group

|  | 9-13 years | |  | 14-18 years | |  | 19-64 years | |  |
| --- | --- | --- | --- | --- | --- | --- | --- | --- | --- |
| **Rank** | **ID** | **Food group** | **Mean choline (mg)** | **ID** | **Food group** | **Mean choline (mg)** | **ID** | **Food group** | **Mean choline (mg)** |
| 1 | 172 | Dishes where egg is the major ingredient | 239.05 | 172 | Dishes where egg is the major ingredient | 156.25 | 182 | Mammalian game meats | 237.31 |
| 2 | 153 | Other sea and freshwater foods | 127.45 | 171 | Eggs | 120.49 | 172 | Dishes where egg is the major ingredient | 232.85 |
| 3 | 171 | Eggs | 104.94 | 189 | Mixed dishes where poultry or feathered game is the major component | 107.75 | 171 | Eggs | 143.67 |
| 4 | 181 | Beef, sheep and pork, unprocessed | 97.85 | 152 | Crustacea and molluscs (excluding commercially sterile) | 107.72 | 187 | Mixed dishes where beef, sheep, pork or mammalian game is the major component | 119.83 |
| 5 | 187 | Mixed dishes where beef, sheep, pork or mammalian game is the major component | 88.97 | 215 | Soup, prepared from condensed or sold ready to heat | 104.14 | 181 | Beef, sheep and pork, unprocessed | 113.30 |
| 6 | 205 | Soy-based yoghurts | 84.70 | 181 | Beef, sheep and pork, unprocessed | 99.82 | 189 | Mixed dishes where poultry or feathered game is the major component | 108.93 |
| 7 | 184 | Organ meats and offal, products and dishes | 84.00 | 183 | Poultry and feathered game | 96.86 | 184 | Organ meats and offal, products and dishes | 101.86 |
| 8 | 189 | Mixed dishes where poultry or feathered game is the major component | 81.13 | 187 | Mixed dishes where beef, sheep, pork or mammalian game is the major component | 88.55 | 156 | Mixed dishes with fish or seafood as the major component | 99.34 |
| 9 | 151 | Fin fish (excluding commercially sterile) | 75.06 | 252 | Mature legume and pulse products and dishes | 84.03 | 183 | Poultry and feathered game | 98.65 |
| 10 | 183 | Poultry and feathered game | 72.37 | 156 | Mixed dishes with fish or seafood as the major component | 79.56 | 155 | Fish and seafood products (homemade and takeaway) | 92.35 |
| 11 | 135 | Mixed dishes where cereal is the major ingredient | 68.98 | 135 | Mixed dishes where cereal is the major ingredient | 74.17 | 151 | Fin fish (excluding commercially sterile) | 90.38 |
| 12 | 156 | Mixed dishes with fish or seafood as the major component | 68.88 | 196 | Custards | 67.34 | 153 | Other sea and freshwater foods | 89.66 |
| 13 | 185 | Sausages, frankfurts (what frankfurters are called in Australia) and saveloys | 67.68 | 301 | Formula dietary foods | 67.05 | 291 | Beers | 75.40 |
| 14 | 251 | Mature legumes and pulses | 62.32 | 198 | Flavoured milks and milkshakes | 64.41 | 185 | Sausages, frankfurts (what frankfurters are called in Australia) and saveloys | 74.31 |
| 15 | 198 | Flavoured milks and milkshakes | 61.19 | 155 | Fish and seafood products (homemade and takeaway) | 56.48 | 205 | Soy-based yoghurts | 71.50 |
| 16 | 301 | Formula dietary foods | 59.73 | 126 | Breakfast cereals, hot porridge style | 56.40 | 152 | Crustacea and molluscs (excluding commercially sterile) | 70.54 |
| 17 | 211 | Soup, homemade from basic ingredients | 57.37 | 251 | Mature legumes and pulses | 54.02 | 135 | Mixed dishes where cereal is the major ingredient | 70.31 |
| 18 | 215 | Soup, prepared from condensed or sold ready to heat | 55.43 | 134 | Pastries | 52.45 | 198 | Flavoured milks and milkshakes | 65.69 |
| 19 | 182 | Mammalian game meats | 54.60 | 151 | Fin fish (excluding commercially sterile) | 51.77 | 188 | Mixed dishes where sausage, bacon, ham or other processed meat is the major component | 59.47 |
| 20 | 126 | Breakfast cereals, hot porridge style | 54.05 | 211 | Soup, homemade from basic ingredients | 48.00 | 216 | Soup, purchased ready to eat | 59.20 |

**Table S4.** Average intakes for pregnant and lactating female consumers of choline by food group

|  | Pregnant females 19+ years | |  | Lactating women | | |
| --- | --- | --- | --- | --- | --- | --- |
| **Rank** | **ID** | **Food group** | **Mean choline (mg)** | **ID** | **Food group** | **Mean choline (mg)** |
| 1 | 153 | Other sea and freshwater foods | 219.78 | 172 | Dishes where egg is the major ingredient | 248.12 |
| 2 | 172 | Dishes where egg is the major ingredient | 183.25 | 171 | Eggs | 157.92 |
| 3 | 216 | Soup, purchased ready to eat | 121.51 | 187 | Mixed dishes where beef, sheep, pork or mammalian game is the major component | 144.53 |
| 4 | 171 | Eggs | 116.19 | 189 | Mixed dishes where poultry or feathered game is the major component | 132.71 |
| 5 | 152 | Crustacea and molluscs (excluding commercially sterile) | 111.71 | 181 | Beef, sheep and pork, unprocessed | 117.18 |
| 6 | 181 | Beef, sheep and pork, unprocessed | 102.84 | 155 | Fish and seafood products (homemade and takeaway) | 97.88 |
| 7 | 151 | Fin fish (excluding commercially sterile) | 99.36 | 151 | Fin fish (excluding commercially sterile) | 81.41 |
| 8 | 185 | Sausages, frankfurts (what frankfurters are called in Australia) and saveloys | 97.33 | 185 | Sausages, frankfurts (what frankfurters are called in Australia) and saveloys | 81.36 |
| 9 | 156 | Mixed dishes with fish or seafood as the major component | 95.46 | 135 | Mixed dishes where cereal is the major ingredient | 79.55 |
| 10 | 189 | Mixed dishes where poultry or feathered game is the major component | 92.00 | 198 | Flavoured milks and milkshakes | 78.49 |
| 11 | 187 | Mixed dishes where beef, sheep, pork or mammalian game is the major component | 86.50 | 196 | Custards | 76.01 |
| 12 | 183 | Poultry and feathered game | 83.78 | 183 | Poultry and feathered game | 74.70 |
| 13 | 135 | Mixed dishes where cereal is the major ingredient | 81.13 | 252 | Mature legume and pulse products and dishes | 63.66 |
| 14 | 201 | Dairy milk substitutes, unflavoured | 76.16 | 211 | Soup, homemade from basic ingredients | 45.87 |
| 15 | 198 | Flavoured milks and milkshakes | 74.49 | 133 | Cakes, muffins, scones, cake-type desserts | 45.61 |
| 16 | 206 | Meat substitutes | 71.98 | 156 | Mixed dishes with fish or seafood as the major component | 44.40 |
| 17 | 134 | Pastries | 50.08 | 191 | Dairy milk (cow, sheep and goat) | 38.22 |
| 18 | 155 | Fish and seafood products (homemade and takeaway) | 48.77 | 134 | Pastries | 37.10 |
| 19 | 126 | Breakfast cereals, hot porridge style | 48.69 | 206 | Meat substitutes | 31.30 |
| 20 | 211 | Soup, homemade from basic ingredients | 48.28 | 216 | Soup, purchased ready to eat | 29.73 |
